# Demographic, Behavioral, and Clinical Characteristics of Persons Seeking Care at Sexually Transmitted Disease Clinics — 14 Sites, STD Surveillance Network, United States, 2010–2018

**DOI:** 10.15585/mmwr.ss7007a1

**Published:** 2021-11-05

**Authors:** Eloisa Llata, Kendra M. Cuffe, Viani Picchetti, Jimmy R. Braxton, Elizabeth A. Torrone

**Affiliations:** 1 Division of STD Prevention, National Center for HIV/AIDS, Viral Hepatitis, STD, and TB Prevention, CDC

## Abstract

**Problem:**

Sexually transmitted diseases (STDs) are a major cause of morbidity in the United States, with an estimated $15.9 billion in lifetime direct medical costs. Although the majority of STDs are diagnosed in the private sector, publicly funded STD clinics have an important role in providing comprehensive sexual health care services, including STD and HIV screening, for a broad range of patients. In certain cases, STD clinics often are the only source of sexual health care for patients, particularly among gay, bisexual, and other men who have sex with men (MSM).

**Period Covered:**

2010–2018.

**Description of the System:**

The STD Surveillance Network (SSuN) is an ongoing sentinel surveillance system for monitoring clinical information among patients attending STD clinics. SSuN is a collaboration of competitively selected state and city health departments that conduct facility-based sentinel surveillance in STD clinics. Information routinely collected through the course of patient encounters is obtained for all patients seeking care in the participating STD clinics. This information includes demographic, behavioral, and clinical characteristics (e.g., STD and HIV tests performed and STD and HIV diagnoses). This report presents 2010–2018 SSuN data from 14 STD clinics in five cities (Baltimore, Maryland; New York City, New York; Philadelphia, Pennsylvania; San Francisco, California; and Seattle, Washington) to describe the patient populations seeking care in these STD clinics. Estimated numbers and percentages of patients receiving selected STD-related health services were calculated for each year by using an inverse variance weighted random-effects model, adjusting for heterogeneity among SSuN jurisdictions. Trends in receipt of selected STD-related health services were examined and included HIV screening after an acute STD diagnosis among persons not previously known to have HIV infection, annual chlamydia screening among adolescent and young females, and extragenital chlamydia and gonorrhea screening among MSM.

**Results:**

During 2010–2018, the total number of annual visits made in the 14 participating STD clinics decreased 29.8% (from 145,728 to 102,275 visits), and the total number of unique patients examined in the clinics decreased 35.1% (from 94,281 to 61,172 patients). Decreases in the number of unique patients occurred both among men who have sex with women only (42.4%; from 37,842 in 2010 to 21,781 in 2018) and among females (51.4%; from 36,485 in 2010 to 17,721 in 2018). The decreases in the number of female patients were observed across all age groups, although they were more pronounced among females aged ≤24 years (66.4%; from 17,721 in 2010 to 5,962 in 2018). In contrast, the number of patients identified as MSM increased 44.0% (from 12,859 in 2010 to 18,512 in 2018), with the greatest increase among MSM aged ≥25 years (58.6%; from 9,918 in 2010 to 15,733 in 2018). Among visits during which an acute STD (defined as chlamydia, gonorrhea, or primary or secondary syphilis) was diagnosed, the percentage of visits during which an HIV test was performed within approximately 14 days of the STD diagnosis increased from 58.2% in 2010 to 70.2% in 2018. Among those patients tested, 1,672 HIV infections were identified, of which 84.0% were among MSM. Among females aged 15–24 years, the percentage screened for chlamydia in any calendar year increased from 88.6% in 2010 to 90.6% in 2018. However, because fewer females aged 15–24 years attended these clinics during the study period, the crude number of adolescent and young females tested for chlamydia decreased from 14,249 in 2010 to 4,507 in 2018. During 2010–2018, the percentage of females retested after their first positive chlamydia diagnosis during the same year ranged from 11.4% to 13.3%. During 2010–2018, the percentage of MSM tested for rectal chlamydia and rectal gonorrhea increased (from 54.7% to 57.8% and from 55.0% to 58.4%, respectively). During the same period, increases were noted in the percentage of MSM with diagnosed rectal chlamydia (from 15.5% in 2010 to 17.7% in 2018) and rectal gonorrhea (from 13.3% in 2010 to 17.1% in 2018). In contrast with pharyngeal chlamydia, pharyngeal gonorrhea screening was more common (from 69.5% in 2010 to 74.6% in 2018), and the percentage positive doubled during the study period (from 7.3% in 2010 to 14.8% in 2018). Pharyngeal chlamydia testing also increased (from 50.3% in 2010 to 72.9% in 2018), with concurrent decreases in positivity (from 4.2% in 2010 to 2.6% in 2018).

**Interpretation:**

During 2010–2018, changes occurred in the demographic composition of patients attending STD clinics participating in SSuN. Understanding trends in the demographic profile of STD patients and services provided can help identify addressable gaps in STD control efforts and direct public health action. Overall, fewer females, especially those aged 15–24 years, accessed care in these STD clinics during the study period. Untreated STDs among adolescent and young females can have serious consequences, including pelvic inflammatory disease and infertility. Additional efforts to monitor where adolescent and young females seek care and to ensure they are receiving quality STD-related health services are needed, especially considering increases in reported cases of STDs among females. Increases in the number of MSM attending STD clinics present a unique opportunity to reach this population with STD and HIV prevention services. Although a large percentage of STD cases are diagnosed outside of STD clinics, publicly funded STD clinics are an important safety-net provider of STD-related health services and provide vital STD-related health services for patient populations at risk for the consequences of STDs and HIV infection.

**Public Health Actions:**

STD-related health services represent effective strategies for preventing STD and HIV transmission and acquisition or STD-related sequelae. Ensuring that all persons receive quality HIV and STD prevention and treatment services is vital for an effective public health approach to reducing STDs. STD clinics provide crucial safety-net services for preventing STD-related morbidity, including timely identification and treatment of curable STDs such as chlamydia, gonorrhea, and syphilis. Increases in the numbers of MSM attending STD clinics participating in SSuN provide additional opportunities for linking patients to high-impact HIV preventive services (e.g., pre-exposure prophylaxis), and the clinics are positioned to facilitate initiation or resumption of treatment among persons living with HIV.

## Introduction

Reported cases of sexually transmitted diseases (STDs) continue to increase, and STDs remain a major cause of morbidity in the United States. In 2018, a record 2.4 million combined cases of chlamydia, gonorrhea, and syphilis were reported to CDC (*1*). Although these STDs are preventable and curable, the majority of infections are not diagnosed or treated (*1*). In addition, STDs are costly, posing an economic strain of $15.9 billion in lifetime direct medical costs in the United States in 2018 (*2*). Because of the potential for devastating sequelae of untreated STDs (e.g., infertility or mother-to-child transmission), all persons should receive quality STD-related preventive services that include screening and diagnostic testing, recommended and timely treatment, and counseling to help prevent further spread of STDs. This is especially crucial for populations vulnerable to STDs, including gay, bisexual, and other men who have sex with men (MSM), adolescent and young females, and persons identifying as transgender (including male-to-female, female-to-male, and transgender unspecified).

To complement other surveillance strategies (e.g., case-based surveillance and population-based surveys), enhanced surveillance in sentinel sites, including STD clinics, provides for monitoring trends among populations at risk for the consequences of STDs and HIV infection. Understanding who seeks care in STD clinics and the patterns of STD-related health services provided by those clinics is important for planning and monitoring STD and HIV prevention and control program activities. In addition, funding priorities have shifted, and a reduction in the number of STD clinics has been observed nationwide, which might have resulted in reduced access to STD-related care for certain populations (*3*–*6*). Understanding trends in the demographic profile of STD patients and services provided can help identify gaps in STD control efforts and direct public health action. This report presents data from STD clinics participating in a sentinel STD surveillance system during 2010–2018, describing trends in selected demographic patient characteristics and selected STD and HIV prevention services. Information about the types of patients who access care in STD clinics and the selected preventive and diagnostic services offered by the clinics is important for program planners and policymakers seeking to ensure that persons in these health care settings receive comprehensive STD and HIV care, especially because of the challenges and changes brought about by transitions in health care financing and delivery since 2010.

## Methods

### Data Source

The STD Surveillance Network (SSuN) comprises state and city health departments that receive funding for facility-based surveillance activities. These departments collect and report to CDC the demographic, behavioral, and clinical data about patients seeking STD-related health services at participating STD clinics. During SSuN cycle 1 (2005–2008), six jurisdictions were funded primarily for conducting genital wart and gonorrhea surveillance. During SSuN cycle 2 (2008–2013), funding was expanded to 12 collaborating state and city health departments, and data collection for facility-based surveillance encompassed the full census of STD clinic patients, not just those with selected diagnoses. SSuN cycle 3 (2013–2018) followed similar methods as cycle 2, continuing the core functions of the network in monitoring the incidence of STDs and care-seeking behaviors among all patients at participating STD clinics. Because cycles 2 and 3 used similar methods, data were included from the overlapping five jurisdictions with 14 STD clinics that participated in SSuN cycles 2 and 3 and that transmitted data for the entire 9 calendar years (2010–2018).

### Participating Jurisdictions and STD Clinics

State and local health departments applied to participate in the SSuN cycles 2 and 3 as part of the funding opportunity announcements STD Surveillance Network CDC-RFA-PS08-865 and STD Surveillance Network CDC-RFA-PS13-1306. Five state and city health departments providing data from 14 STD clinics submitted SSuN facility data during 2010–2018: Baltimore, Maryland (two clinics); New York City, New York (eight clinics); Philadelphia, Pennsylvania (two clinics); San Francisco, California (one clinic); and Seattle, Washington (one clinic). SSuN is not designed to be nationally representative; rather, sites are competitively selected to fulfill the objectives and scope of the project across multiple geographic settings as outlined by the notices of funding opportunity. The 14 STD clinics in the five cities represent clinics with large numbers of patients, including MSM, adolescents, and young adults.

### Data Collection

Visit-level demographic, behavioral, and clinical data were collected from the full census of patients seeking services in participating SSuN STD clinics as documented in each clinic’s electronic medical records; these data also were collated across patient visits each year. Clinic visits that were classified as express visits (i.e., triage-based STD screening visits for asymptomatic patients whose history was negative for risk factors and who did not receive a comprehensive examination) were not included in this analysis. The term “visit” is defined as a single nonexpress visit to the STD clinic. For cycles 2 and 3, SSuN collaborators in each of the funded jurisdictions and CDC’s principal investigators defined data elements. All the data collected for this project were ascertained through routinely collected electronic health records from a clinic visit. The investigators did not conduct patient interviews. Extracted data for this analysis included age, race/ethnicity, sex/gender identity, sex of sex partners, HIV status, previous HIV screening and results, STD diagnoses, and laboratory screening and results. 

Unique patient and clinic or event identifiers were established to provide longitudinal monitoring of multiple visits by patients within each clinic. SSuN jurisdictions, in conjunction with data management staff members at the participating STD clinics, produced three main data sets. The first of these was a visit file containing demographic, behavioral, and self-reported HIV infection status information collected at that visit. The second file contained visit-level STD-related diagnoses. The third file contained visit-level laboratory tests and results. Participating SSuN jurisdictions completed data verification and validity checks on each of the files and transmitted deidentified data to CDC through a secure data portal. When CDC received the files, additional data verification and validity checks for quality assurance, including consistency checks to ensure that data in the records were internally rational, were performed. All three files were linked by the unique patient and visit or event identifiers allowing for deduplication. Errors, including duplicate patient or event identifiers, were submitted to the jurisdictions, and the jurisdictions were asked to fix the errors before retransmission to CDC. 

After the processing of data, the files were merged into the national SSuN data sets for analysis. Jurisdictions upheld the Data Security and Confidentiality Guidelines for HIV, Viral Hepatitis, Sexually Transmitted Diseases, and Tuberculosis Programs (*7*). CDC’s Human Research Protection Office reviewed the SSuN protocol and deemed it to be a surveillance and disease control activity. Analysis of deidentified SSuN data does not constitute research involving human subjects; therefore, Institutional Review Board review was not required. 

### Data Analysis

Annual counts and percentages were calculated to describe patient characteristics for those who had used each STD clinic in each participating jurisdiction and receipt of the following selected recommended STD-related health services: 1) HIV screening among persons not known to be living with diagnosed HIV infection who received a diagnosis of an acute STD, 2) chlamydia screening among adolescent and young females and rescreening at 8–16 weeks among those who had a documented chlamydial infection, and 3) extragenital chlamydia and gonorrhea screening among MSM. Annual counts were based on either the number of unique patients or the number of visits, depending on the selected characteristic being analyzed.

The estimated percentages (e.g., percentage of MSM tested for extragenital gonorrhea) might have varied across clinics, and simply aggregating the data across jurisdictions could lead to biased estimates if heterogeneity were not considered. In addition, clinics provided data for a full census of patients, and simple aggregation would be biased toward clinics with the largest numbers of patients. Therefore, for each of the demographic and STD-related health service estimates, jurisdiction-specific estimates for each year were calculated and combined by using an inverse variance weighted random-effects model. The random-effects model incorporated heterogeneity across the jurisdictions by allowing each jurisdiction to have a different true prevalence. SAS (version 9.4; SAS Institute) was used to conduct all analyses.

#### Patient Demographics

Trends in five demographic and clinical characteristics of patients were described: sex/gender identity, race/ethnicity, age, sex of sex partners, and HIV status. Sex/gender identity categories were male, female, transgender, and unknown. Race/ethnicity was categorized as non-Hispanic (NH) White, NH Black, Hispanic, NH Asian/Native Hawaiian and other Pacific Islander (NHOPI), or NH other (includes American Indian/Alaska Native, multiple race, and unknown race). Age was based on age at last clinic visit of each year and was grouped into categories of ≤24 years and ≥25 years, except for the analysis of chlamydia screening (i.e., only females aged 15–24 years were analyzed). Patients were categorized as MSM, men who have sex with women only (MSW), females, and males with unknown sex of sex partners. Classification of male sexual behavior/orientation was based on the sex partner’s reported or self-identification of sexual orientation as reported by the patient. Sexual orientation and sex of sex partner data were collected for female patients, and the majority identified as heterosexual women and reported only sex with men. However, due to small sample sizes among the female groups who identified as homosexual or bisexual or among those with missing information, results stratified by sexual orientation were not shown. Hence, females without regard to the sex of their sex partners were reported. Patients were categorized as known to be living with diagnosed HIV infection if a laboratory-documented positive HIV antibody test was included in their clinic records or the record contained a self-reported HIV diagnosis. All other patients were categorized as HIV uninfected or status unknown.

#### HIV Screening

The U.S. Preventive Services Task Force (USPSTF) recommends routine HIV screening among all persons requesting testing for STDs (*8*). All visits during 2010–2018 in which a diagnosis of chlamydia, gonorrhea, or primary or secondary syphilis was made (referred to in this report as acute STD diagnoses) were included. To characterize HIV screening among patients with STD diagnoses, distinct episodes were identified, which included an index visit (i.e., a visit in which the patient received an acute STD diagnosis) plus any return visits within 30 days most likely related to the index visit. Return STD clinic visits during which patients were diagnosed with an acute STD after 30 days were likely unrelated and counted as a new episode (*9*). Hence, a patient could have multiple index visits within a calendar year. Patients who were already known to have HIV infection (either by self-report or a previous positive HIV test performed in the clinic) at the time of their acute STD diagnosis were excluded from the analysis. The percentage of visits during which HIV screening occurred (within ≤14 days of the index visit) for each episode and the percentage of positive HIV tests were calculated. To allow for the 14-day screening window, index visits were restricted to January 1–December 17 of each calendar year.

#### Chlamydia Screening Among Females Aged 15–24 Years 

Because the majority of chlamydial infections among women are asymptomatic, USPSTF recommends that all sexually active females aged ≤24 years be screened for chlamydia (*10*). In addition, the CDC *Sexually Transmitted Infections Treatment Guidelines, 2021*, encourage rescreening at 3 months after a chlamydia diagnosis to identify and manage reinfections (*11*). Although the guidance is recommended for all sexually active females aged ≤24 years, the highest burden of chlamydia is among females aged 15–24 years. Hence, laboratory data for chlamydia tests performed during January 2010–December 2018 were reviewed for females aged 15–24 years. Chlamydia screening coverage was defined as the number of unique female patients aged 15–24 years screened for chlamydia one or more times during a calendar year divided by the total number of unique female patients aged 15–24 years seeking care that calendar year. Chlamydia positivity was calculated by the number of females aged 15–24 years with at least one positive chlamydia test divided by the number of females aged 15–24 years tested for chlamydia at least once during the calendar year. The percentage of adolescent and young females rescreened for chlamydia in each calendar year was calculated as the number of females aged 15–24 years who had a repeat chlamydia test within 8–16 weeks of their initial positive chlamydia test divided by the total number of females aged 15–24 years who had an initial positive chlamydia test. Tracking unique patients across years was not possible because the assignment of unique patient identifiers was not maintained across years in certain participating jurisdictions. Chlamydia repeat positivity was calculated as the percentage of females aged 15–24 years who had positive tests at rescreening divided by the number of females aged 15–24 years rescreened. For the rescreening percentage estimation, the analysis was limited to females aged 15–24 years who had positive tests during the first 8 months (January 1–August 31) of each calendar year to allow for 8–16 weeks of follow-up time for each female. If females had multiple visits with a positive chlamydia test each year, rescreening rates were assessed by using the first visit with a positive chlamydia test in that year. Differences in screening and positivity by age group and race/ethnicity were assessed. However, the percentage rescreened and repeat positivity could not be stratified by demographic characteristics because of small sample sizes. Differentiating between asymptomatic screening and diagnostic testing was not possible because a substantial portion of visits were missing symptom status in the SSuN data files. Consequently, screening and rescreening rates likely included patients who were tested to detect symptomatic disease and patients screened to detect asymptomatic infections.

#### Extragenital (Rectal and Pharyngeal) Chlamydia and Gonorrhea Screening Among MSM

CDC recommends that all MSM who have anal intercourse be screened annually for rectal chlamydia and gonorrhea and that all MSM who have receptive oral intercourse be screened for pharyngeal gonorrhea (*11*). Rectal gonorrhea screening coverage was estimated as the percentage of MSM who had received a rectal gonorrhea test during a calendar year divided by the total number of MSM seeking care that calendar year. Rectal gonorrhea positivity was estimated as the number of MSM with at least one positive rectal gonorrhea test result divided by the number of MSM with at least one rectal gonorrhea test that year. Screening coverage and positivity also were calculated for chlamydia. Trend data for pharyngeal chlamydia screening among MSM only included data from SSuN clinics in Baltimore, Philadelphia, San Francisco, and Seattle. Information about anatomic site of sexual exposure was not transmitted to CDC consistently during the entire study period. Hence, the trend data should be viewed as a description of how extragenital screening and positivity have changed temporally and not as an evaluation of the compliance of extragenital screening on the basis of sexual exposure history. (For example, certain MSM included in the denominator of the rectal or pharyngeal screening coverage estimates might not have had receptive anal or oral intercourse.) As with estimates of chlamydia screening among young females, a lack of data regarding symptom status prohibited the ability to differentiate asymptomatic screening from diagnostic testing. Consequently, screening estimates included all MSM who were screened and diagnostically tested regardless of symptoms.

## Results

### Patient Demographics by Gender Identity and Selected Sexual Behavior/Orientation Characteristics 

**FIGURE 1:**
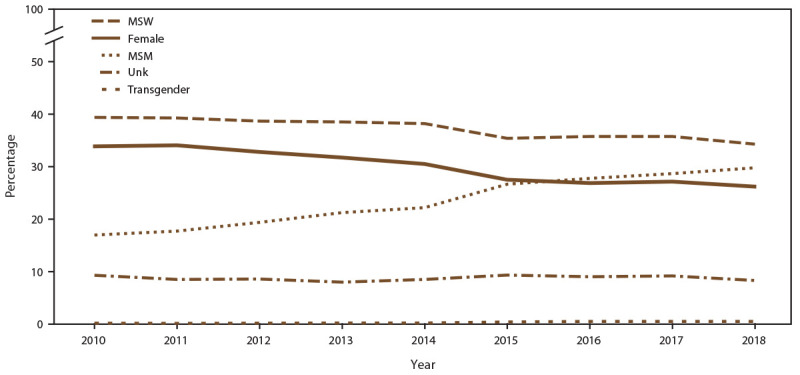
Percentage of unique patients, by gender identity and selected sexual behavior/orientation characteristics* and year — STD Surveillance Network, 14 sites, United States, 2010–2018 **Abbreviations:** MSM = gay, bisexual, and other men who have sex with men; MSW = men who have sex with women only; STD = sexually transmitted disease; Unk = unknown sex of sex partner. * Transgender includes male-to-female transgender, female-to-male transgender, and transgender unspecified.

During 2010–2018, a total of 713,380 unique patients sought care at 14 STD clinics from five jurisdictions with continuous participation in SSuN during this time frame (accounting for 1,123,594 nonexpress clinic visits) (Table). The number of unique patients and visits varied across SSuN jurisdictions. New York City had the highest total number of patients and visits (346,670 patients and 498,173 visits), and Seattle had the lowest number of patients and visits (62,523 patients and 99,603 visits). Overall, the number of unique patients seeking care in the participating STD clinics decreased (35.1%; from 94,281 patients in 2010 to 61,172 patients in 2018), with concurrent decreases in the number of total patient visits (29.8%; from 145,728 in 2010 to 102,275 visits in 2018). When stratified by gender identity and selected sexual behavior/orientation characteristics, declines were more pronounced among females when compared with MSW or transgender persons (Figure 1). Decreases in unique patients and visits were observed in all jurisdictions, although at different rates. For example, the number of unique patients in participating SSuN clinics during 2010–2018 decreased 42.7% in New York City and 23.1% in San Francisco, and the number of visits decreased 53.2% in Baltimore but only 5.3% in Seattle (Table). Trends in the percentages of patients by race/ethnicity did not vary substantially throughout the 9-year period (Supplementary Figure 1, https://stacks.cdc.gov/view/cdc/110781). However, fluctuations were noted in trends in unique patients by age group. For example, decreases occurred in the percentages of patients aged ≤24 years (33.6% in 2010 versus 21.3% in 2018), concurrent with increases in the percentage of patients aged ≥25 years (66.4% in 2010 versus 78.7% in 2018) (Supplementary Figure 2, https://stacks.cdc.gov/view/cdc/110781). Overall, the number of patients known to be living with diagnosed HIV infection who were accessing care in participating SSuN clinics increased (8.8%; from 3,535 in 2010 to 3,845 in 2018), whereas the number of visits decreased (6.6%; from 8,545 in 2010 to 7,981 in 2018).

**TABLE:** Number of unique patients and clinic visits,* by jurisdiction and year seeking care in sexually transmitted disease clinics — STD Surveillance Network, 14 sites, United States, 2010–2018

Year	Baltimore, Maryland (2 clinics)		Seattle, Washington (1 clinic)		Philadelphia, Pennsylvania (2 clinics)		New York City, New York (8 clinics)		San Francisco, California (1 clinic)		Total (14 clinics)	
	No. of patients	No. of visits	No. of patients	No. of visits	No. of patients	No. of visits	No. of patients	No. of visits	No. of patients	No. of visits	No. of patients	No. of visits
2010	14,185	26,863	8,308	12,773	14,030	23,506	46,462	64,155	11,296	18,431	**94,281**	**145,728**
2011	13,544	25,553	7,634	11,712	13,953	23,763	55,207	75,062	11,782	18,670	**102,120**	**153,760**
2012	12,569	23,447	7,659	11,640	13,087	23,235	52,842	71,551	11,150	18,205	**97,307**	**148,078**
2013	11,846	21,199	7,331	11,247	13,651	23,791	47,845	66,537	10,982	18,902	**91,655**	**141,676**
2014	9,605	17,394	6,498	9,884	13,767	23,913	42,969	58,990	10,894	17,883	**83,733**	**128,064**
2015	7,462	11,881	6,569	9,306	13,427	23,557	25,465	38,035	10,583	18,516	**63,506**	**101,295**
2016	6,891	11,039	6,129	10,077	12,998	23,107	23,771	39,061	9,786	17,658	**59,575**	**100,942**
2017	6,981	11,550	6,017	10,869	12,336	20,545	25,509	41,709	9,188	17,103	**60,031**	**101,776**
2018	7,572	12,579	6,378	12,095	11,936	18,203	26,600	43,073	8,686	16,325	**61,172**	**102,275**
% change from 2010 to 2018	−46.6%	−53.2%	−23.2%	−5.3%	−14.9%	−22.6%	−42.7%	−32.9%	−23.1%	−11.4%	**−35.1%**	**−29.8%**

**Abbreviation:** STD = sexually transmitted disease.

* Visits pertain to nonexpress visits that included comprehensive history and physical examination and laboratory testing. Express clinic visits, classified as triage-based STD screening visits for asymptomatic patients whose history was negative for risk factors and who did not receive a comprehensive examination, were not included.

#### MSM

**FIGURE 2:**
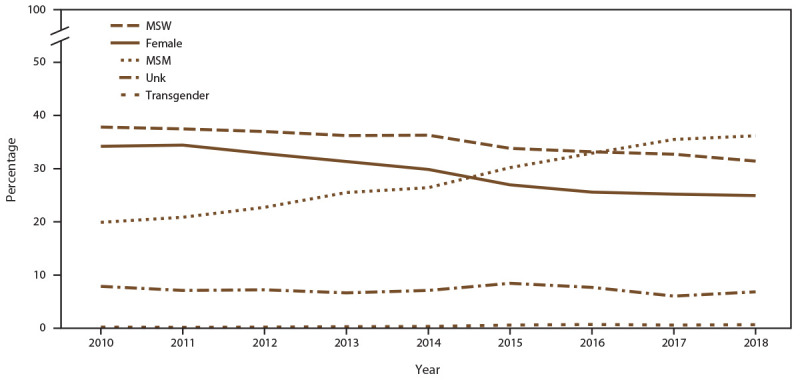
Percentage of patient visits, by gender identity and selected sexual behavior/orientation characteristics* and year — STD Surveillance Network, 14 sites, United States, 2010–2018 **Abbreviations:** MSM = gay, bisexual, and other men who have sex with men; MSW = men who have sex with women only; STD = sexually transmitted disease; Unk = unknown sex of sex partner. * Transgender includes male-to-female transgender, female-to-male transgender, and transgender unspecified.

The number of unique MSM patients seeking care at the 14 participating STD clinics increased 44.0% (from 12,859 in 2010 to 18,512 in 2018) (Supplementary Table 1, https://stacks.cdc.gov/view/cdc/110780). In addition, clinic visits by MSM increased 76.3% (Figure 2) (Supplementary Table 1, https://stacks.cdc.gov/view/cdc/110780). During 2010, unique MSM patients accounted for 17.9% of the clinic populations; however, by 2018, MSM accounted for 30.9%. (Figure 1) (Supplementary Table 2, https://stacks.cdc.gov/view/cdc/110780). During 2010–2018, the percentage of MSM who were NH Black decreased (from 38.8% in 2010 to 29.0% in 2018), as did the percentage who were NH White (from 37.5% in 2010 to 33.1% in 2018) (Supplementary Table 3, https://stacks.cdc.gov/view/cdc/110780). Concurrent increases occurred in the percentages of MSM who were Hispanic (from 14.8% in 2018 to 20.7% in 2019 ), NH Asian/NHOPI (from 5.1% in 2010 to 6.5% in 2018), and NH other race (from 3.8% in 2010 to 10.7% in 2018). Among MSM, the percentage aged ≥25 years increased (from 77.1% in 2010 to 85.0% in 2018) (data not shown). The number of MSM visiting participating clinics who were known to be living with diagnosed HIV infection increased (from 2,520 in 2010 to 3,190 in 2018), although the overall percentage of MSM who were known to be living with diagnosed HIV infection decreased (from 22.5% in 2010 to 17.7% in 2018) (Supplementary Table 4, https://stacks.cdc.gov/view/cdc/110780).

#### MSW

During 2010–2018, the number of unique MSW patients seeking care at the 14 participating STD clinics decreased (42.4%; from 37,842 in 2010 to 21,781 in 2018), as did the number of clinic visits by MSW (Figure 2) (Supplementary Table 1, https://stacks.cdc.gov/view/cdc/110780). Overall, the percentage of patients who were MSW decreased from 39.4% in 2010 to 34.3% in 2018 (Figure 1) (Supplementary Table 1, https://stacks.cdc.gov/view/cdc/110780). Among MSW, the percentages decreased among those who were NH White (from 25.2% in 2010 to 20.5% in 2018) and NH Black (from 54.8% in 2010 to 50.8% in 2018). Concurrent increases occurred in the percentages of MSW who were Hispanic (from 12.2% in 2010 to 13.5% in 2018), NH Asian/NHOPI (from 3.6% in 2010 to 5.0% in 2018), and NH other race (from 4.2% in 2010 to 10.2% in 2018). During 2010–2018, the percentage of MSW aged <25 years decreased from 28.8% in 2010 to 18.8% in 2018. The percentage of MSW patients who were known to be living with diagnosed HIV infection visiting participating SSuN clinics was stable (1.1% in 2010 versus 1.0% in 2018) (Supplementary Table 4, https://stacks.cdc.gov/view/cdc/110780).

#### Males with Unknown Sex of Sex Partners

During 2010–2018, the number of unique male patients with unknown sex of sex partners seeking care at the 14 participating STD clinics decreased 59.9% (from 6,962 patients in 2010 to 2,794 patients in 2018). Clinic visits made by males with unknown sex of sex partners also decreased (Figure 2) (Supplementary Table 1, https://stacks.cdc.gov/view/cdc/110780). Overall, the percentage of males with unknown sex of sex partners visiting participating clinics increased, from 4.8% in 2010 to 5.2% in 2018 (Supplementary Table 2, https://stacks.cdc.gov/view/cdc/110780).

#### Females

During 2010–2018, the number of unique female patients seeking care at the 14 participating STD clinics decreased (51.4%; from 36,485 patients in 2010 to 17,721 patients in 2018), as did the clinic visits by females (Figure 2) (Supplementary Table 1, https://stacks.cdc.gov/view/cdc/110780). Overall, females accounted for a decreasing percentage of all patients (from 34.7% in 2010 to 26.4% in 2018) (Figure 1) (Supplementary Table 2, https://stacks.cdc.gov/view/cdc/110780). Among females, the percentages of those who were NH Black and NH White decreased (from 54.2% and 21.6% in 2010 to 49.3% and 19.4% in 2018, respectively). Increases occurred in the percentages of females who were Hispanic (from 12.0% in 2010 to 13.9% in 2018), NH Asian/NHOPI (from 6.3% in 2010 to 7.1% in 2018), and NH other race (from 5.9% in 2010 to 10.3% in 2018). Among females, the percentage of those aged ≤24 years decreased (from 44.8% in 2010 to 30.4% in 2018 [data not shown]). The percentage of female patients visiting participating SSuN clinics who were known to be living with diagnosed HIV infection remained stable (0.9% in 2010 and 1.0% in 2018) (Supplementary Table 4, https://stacks.cdc.gov/view/cdc/110780).

#### Transgender Persons

The number of unique transgender patients seeking care at the 14 participating STD clinics increased (from 123 patients in 2010 to 299 patients in 2018), with a corresponding increase in the number of clinic visits by transgender patients (from 301 visits in 2010 to 662 visits in 2018) (Figure 2) (Supplementary Table 1, https://stacks.cdc.gov/view/cdc/110780). Overall, among all patients, the percentage of transgender patients increased (from 0.2% in 2010 to 0.6% in 2018) (Supplementary Table 2, https://stacks.cdc.gov/view/cdc/110780). Among transgender patients, decreases occurred in the percentages who were NH Black (from 38.1% in 2010 to 21.1% in 2018) and NH White (from 26.6% in 2010 to 22.8% in 2018). During 2010–2018, concurrent increases occurred in the percentages of transgender patients who were Hispanic (from 26.8% in 2010 to 31.3% in 2018), NH Asian/NHOPI (from 5.7% in 2010 to 7.8% in 2018), and NH other race (from 2.8% in 2010 to 17.0% in 2018). When stratified by age group, the percentage of transgender patients aged ≤24 years decreased (from 25.1% in 2010 to 18.1% in 2018). The percentage of transgender patients visiting participating clinics who were known to be living with diagnosed HIV infection increased (from 10.9% in 2010 to 13.0% in 2018) (Supplementary Table 4, https://stacks.cdc.gov/view/cdc/110780).

### Selected STD-Related Health Services

#### HIV Screening

**FIGURE 3:**
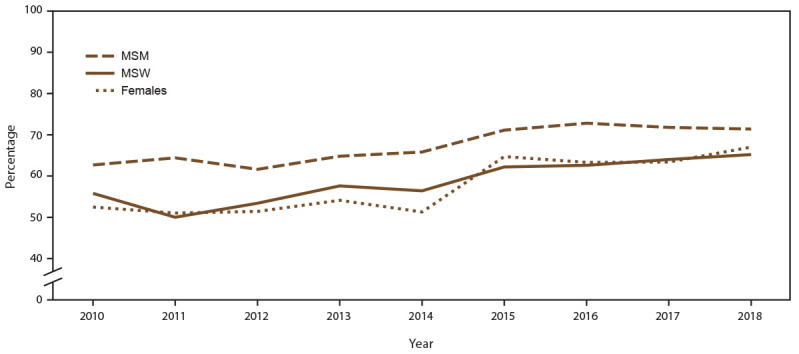
Percentage of sexually transmitted disease clinic visits by patients with a diagnosed acute sexually transmitted disease* who were screened for HIV,^†^ by gender identity and selected sexual behavior/orientation characteristics and year — STD Surveillance Network, 14 sites, United States, 2010–2018 **Abbreviations:** MSM = gay, bisexual, and other men who have sex with men; MSW = men who have sex with women only; STD = sexually transmitted disease. * Acute STD diagnosis is defined as a diagnosis of chlamydia, gonorrhea, or primary or secondary syphilis. ^†^ Excludes all patients who were known to be living with diagnosed HIV infection, either by laboratory documentation or self-report of HIV infection, before the visit during which an acute STD diagnosis was made.

During 2010–2018, the overall percentage of unique patients with a diagnosed acute STD (defined in this report as chlamydia, gonorrhea, or primary or secondary syphilis) at least once during the calendar year increased (from 9.0% in 2010 to 12.9% in 2018), although the absolute number of clinic patients with at least one acute STD diagnosis annually decreased (from 11,460 patients in 2010 to 10,098 patients in 2018). Overall, the percentage of patients with an acute STD diagnosis who were not known to be living with diagnosed HIV infection screened for HIV within ≤14 days of the STD diagnosis increased (from 58.2% in 2010 to 70.2% in 2018). HIV screening among persons with an acute STD diagnosis increased for MSM, MSW, and females (Supplementary Table 5, https://stacks.cdc.gov/view/cdc/110780). However, HIV screening coverage was higher among MSM than among MSW and females throughout the period (Figure 3). Among patients screened for HIV, a total of 1,672 infections were identified (overall positivity: 1.7%), of which 84.0% were among MSM. HIV screening coverage also varied by specific diagnosed STD. For example, HIV screening coverage among MSM with diagnosed rectal gonorrhea ranged from 65.5% in 2010 to 70.4% in 2018 compared with HIV screening among MSM with diagnosed primary and secondary syphilis, ranging from 55.9% in 2010 to 65.8% in 2018 (Supplementary Table 6, https://stacks.cdc.gov/view/cdc/110780).

#### Chlamydia Screening Among Females Aged 15–24 Years

**FIGURE 4:**
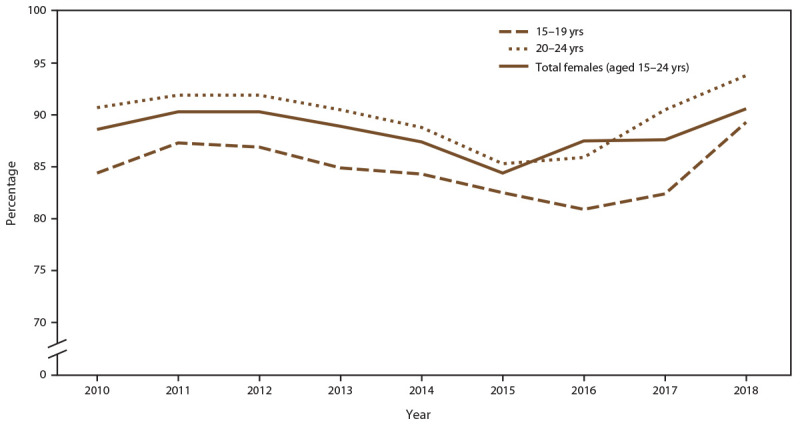
Percentage of females aged 15–24 years receiving chlamydia screening tests at least annually, by age group and year — STD Surveillance Network, 14 sites, United States, 2010–2018 **Abbreviation:** STD = sexually transmitted disease.

**FIGURE 5:**
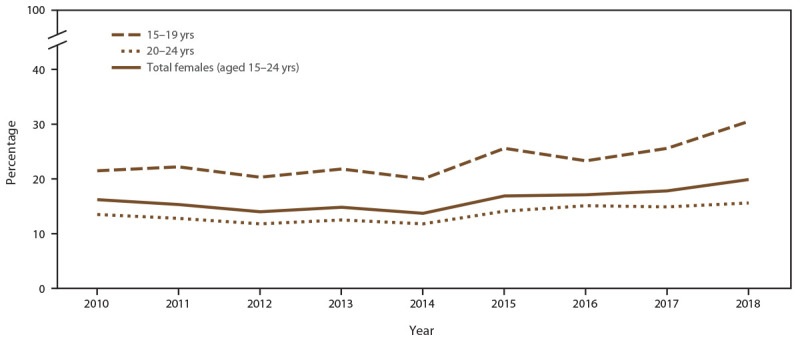
Percentage of females aged 15–24 years with at least one positive chlamydia test, by age group and year — STD Surveillance Network, 14 sites, United States, 2010–2018 **Abbreviation:** STD = sexually transmitted disease.

The percentage of females aged 15–24 years screened for chlamydia at least once during a calendar year was 88.6% in 2010 and 90.6% in 2018) (Figure 4) (Supplementary Table 7, https://stacks.cdc.gov/view/cdc/110780). The number of females screened for chlamydia decreased during the study period because of decreases in the number of adolescent and young females seeking care in the participating 14 clinics. In 2010, a total of 14,249 females aged 15–24 years were screened for chlamydia, compared with 4,507 screened in 2018, representing a 68.4% decrease throughout the study period. The overall percentage of females with at least one positive chlamydia test during any calendar year increased (from 16.2% in 2010 to 19.9% in 2018) (Figure 5) (Supplementary Table 7, https://stacks.cdc.gov/view/cdc/110780).

When stratified by age group, the percentage of females aged 15–19 years screened for chlamydia each year increased from 84.4% in 2010 to 89.3% in 2018 (Figure 4) (Supplementary Table 7, https://stacks.cdc.gov/view/cdc/110780). Higher chlamydia screening coverage was observed among females aged 20–24 years (from 90.7% in 2010 to 93.8% in 2018). When stratified by age group, females aged 15–19 years (from 21.5% in 2010 to 30.5% in 2018) had consistently higher positivity than those aged 20–24 years (from 13.5% in 2010 to 15.6% in 2018) (Figure 5).

**FIGURE 6:**
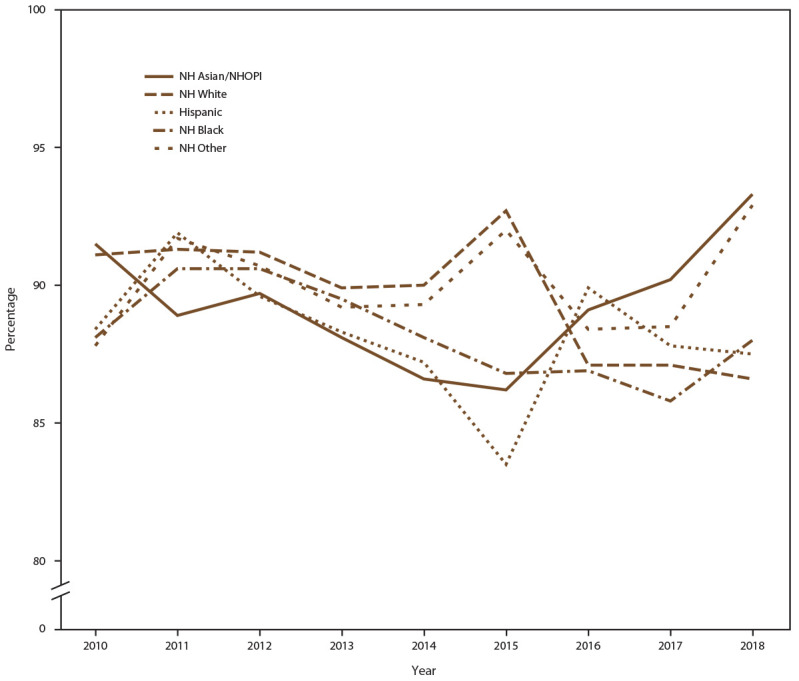
Percentage of females aged 15–24 years receiving chlamydia screening tests at least annually, by race/ethnicity* and year — STD Surveillance Network, 14 sites, United States, 2010–2018. **Abbreviations:** NH = non-Hispanic; NH Asian/NHOPI = non-Hispanic Asian/Native Hawaiian and other Pacific Islander; STD = sexually transmitted disease. * Other includes American Indian/Alaska Native, multiple race, and unknown race.

**FIGURE 7:**
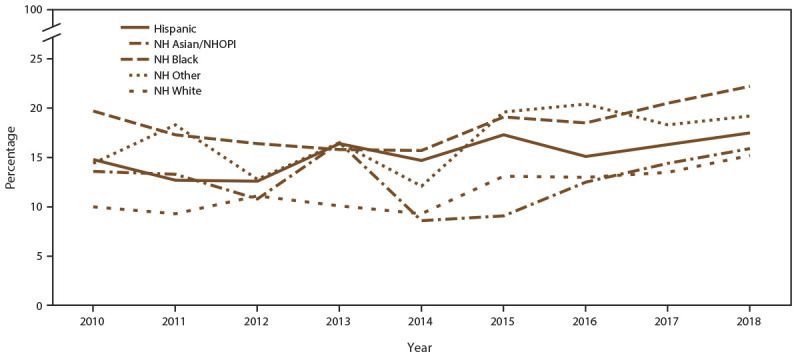
Percentage of females aged 15–24 years with at least one positive chlamydia test, by race/ethnicity* and year — STD Surveillance Network, 14 sites, United States, 2010–2018 **Abbreviations:** NH = non-Hispanic; NH Asian/NHOPI = non-Hispanic Asian/Native Hawaiian and other Pacific Islander; STD = sexually transmitted disease. * Other includes American Indian/Alaska Native, multiple race, and unknown race.

When stratified by race/ethnicity, the percentage screened for chlamydia each year ranged from 80% to 93% in all racial/ethnic groups during the study period (Figure 6) (Supplementary Table 8, https://stacks.cdc.gov/view/cdc/110780). However, chlamydia positivity varied, with higher positivity among females who were NH Black and NH other race noted across all years, compared with females who were NH White, Hispanic, or NH Asian/NHOPI (Figure 7) (Supplementary Table 8, https://stacks.cdc.gov/view/cdc/110780).

**FIGURE 8:**
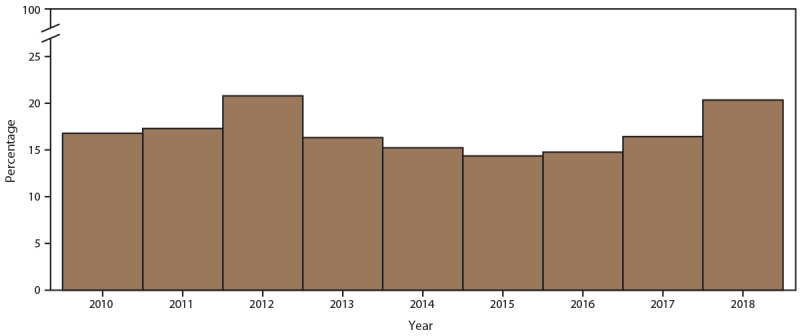
Percentage of females aged 15–24 years who were rescreened for chlamydia 8–16 weeks after a chlamydia diagnosis, by year — STD Surveillance Network, 14 sites, United States, 2010–2018 **Abbreviation:** STD = sexually transmitted disease.

Rescreening at 8–16 weeks among adolescent and young females after their first positive chlamydia test varied by year (Figure 8). In 2010, 11.4% of females were rescreened upon returning during the time frame; this increased to 13.3% in 2018 (Supplementary Table 9, https://stacks.cdc.gov/view/cdc/110780). Each year, rescreening positivity (23.0% in 2010 versus 27.1% in 2018) was higher than overall screening positivity (16.2% in 2010 versus 19.9% in 2018). Because of the small sample size, stratification by age group or race/ethnicity among females who were rescreened was not performed.

#### Extragenital (Rectal and Pharyngeal) Chlamydia and Gonorrhea Screening Among MSM

**FIGURE 9:**
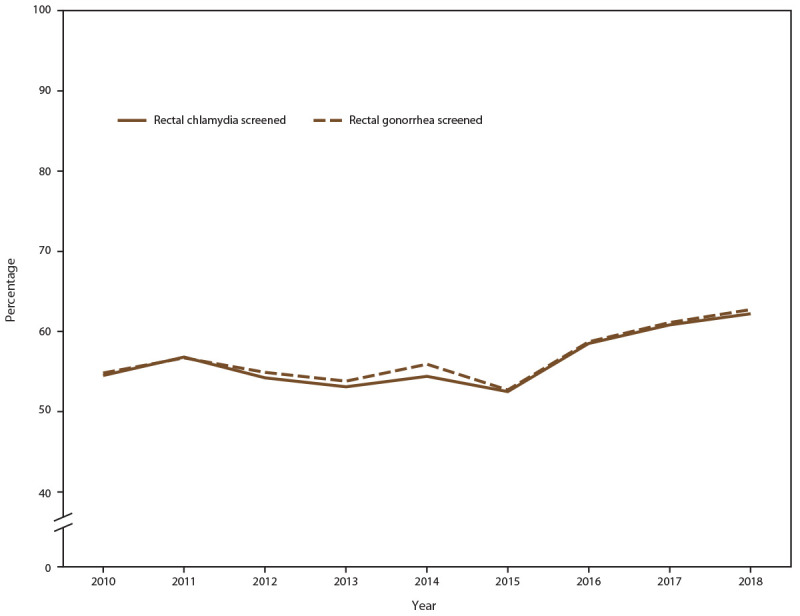
Percentage of gay, bisexual, and other men who have sex with men receiving rectal chlamydia and gonorrhea screening tests at least annually, by year — STD Surveillance Network, 14 sites, United States, 2010–2018 **Abbreviation:** STD = sexually transmitted disease.

**FIGURE 10:**
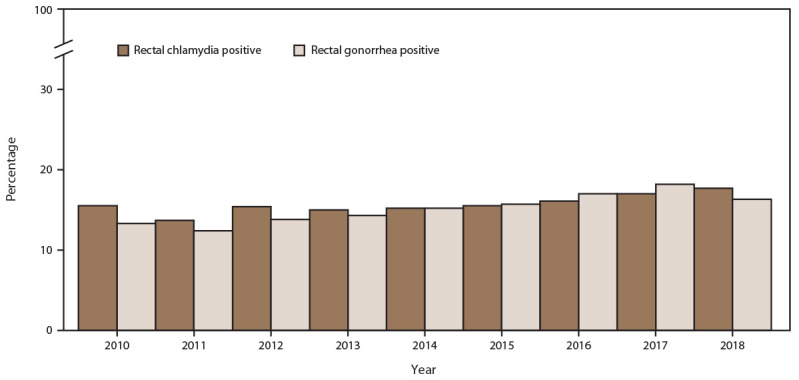
Percentage of gay, bisexual, and other men who have sex with men with positive rectal chlamydia and gonorrhea screening tests, by year — STD Surveillance Network, 14 sites, United States, 2010–2018 **Abbreviation:** STD = sexually transmitted disease.

During 2010–2018, among MSM seeking care at participating STD clinics, the percentage screened for rectal chlamydia at least once annually increased from 54.7% in 2010 to 57.8% in 2018 (Figure 9) (Supplementary Table 10, https://stacks.cdc.gov/view/cdc/110780). Because gonococcal and chlamydial infections are usually detected using a single diagnostic nucleic acid amplification test (NAAT), a similar increase was noted among those screened for rectal gonorrhea (from 55.0% in 2010 to 58.4% in 2018). Absolute numbers of MSM screened increased for both rectal chlamydia (from 7,658 in 2010 to 11,443 in 2018) and rectal gonorrhea (from 7,701 in 2010 to 11,584 in 2018). Throughout the study period, positivity among MSM also increased for rectal chlamydia (from 15.5% in 2010 to 17.7% in 2018) and rectal gonorrhea (from 13.3% in 2010 to 17.1% in 2018) (Figure 10).

**FIGURE 11:**
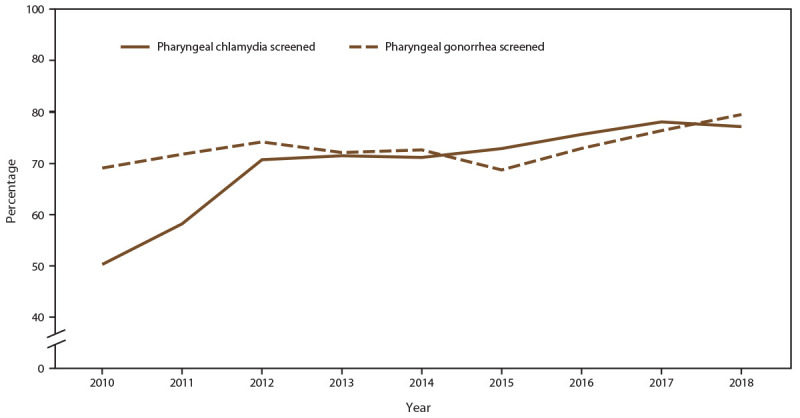
Percentage of gay, bisexual, and other men who have sex with men receiving pharyngeal chlamydia and gonorrhea screening tests at least annually, by year — STD Surveillance Network, 14 sites, United States, 2010–2018 **Abbreviation:** STD = sexually transmitted disease.

**FIGURE 12:**
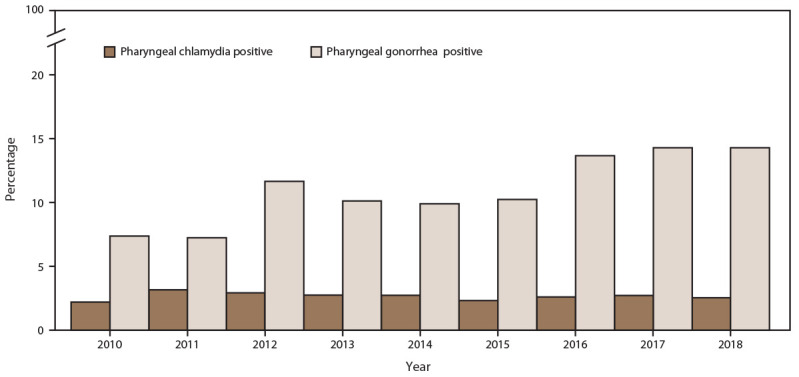
Percentage of gay, bisexual, and other men who have sex with men with positive pharyngeal chlamydia and gonorrhea screening tests, by year — STD Surveillance Network, 14 sites, United States, 2010–2018 **Abbreviation:** STD = sexually transmitted disease.

The percentage of MSM screened for pharyngeal gonorrhea at least once during the calendar year increased (from 69.5% in 2010 to 74.6% in 2018), with a corresponding increase in the annual number of MSM patients screened (45.6%; from 9,834 in 2010 to 14,317 in 2018) (Figure 11) (Supplementary Table 11, https://stacks.cdc.gov/view/cdc/110780). During the same period, pharyngeal gonorrhea positivity doubled among MSM (from 7.3% in 2010 to 14.8% in 2018). Pharyngeal chlamydia screening among MSM also increased (from 50.3% in 2010 to 72.9% in 2018). Pharyngeal chlamydia positivity decreased during the study period (from 4.2% in 2010 to 2.6% in 2018) (Figure 12).

## Discussion

### Patient Demographics

Historically, publicly funded STD clinics have served as a crucial safety-net provider for certain populations (e.g., uninsured persons, racial/ethnic minorities, and MSM) (*12**,**13*). However, patient characteristics and demographics at STD clinics are evolving. Among STD clinics participating in SSuN, the number of unique patients and patient visits decreased during 2010–2018. Identifying and assessing the factors that might have led to this decrease is beyond the scope of this report. However, the decreases might be a result of a combination of factors, including reductions in budgets at state and local health departments leading to less funding for STD-related health services delivery (*14**,**15*), passage and implementation of the Patient Protection and Affordable Care Act (ACA) expanding access to health insurance coverage and health care options (*16**–**18*), and an expanded role for non-STD clinics in diagnosing STDs. An important observation is the increasing trend in the percentages of MSM and transgender persons seeking care in STD clinics across participating jurisdictions. Although the cause of this shift is not fully understood, these populations might regard these STD clinics as providers of culturally competent, confidential, and expert sexual health information (*12*). One study reported that MSM accounted for the highest percentage of insured patients at publicly funded clinics and that MSM populations might feel stigmatized in primary care provider settings (*19*). Although a small percentage of the total patient population, the overall patient volume and number of clinic visits also have increased among transgender persons. Studies have elucidated poorer health and socioeconomic outcomes for transgender populations because of stigma, lack of culturally competent health care providers, lack of transportation, and cost-related barriers (*20*,*21*). Because of the sexual health expertise in STD clinics, transgender persons might encounter providers trained in transgender health who have greater sensitivity regarding concerns of the transgender population. However, research is needed to determine where transgender populations seek STD-related health services and the reasons for selecting those facilities.

Although a substantial proportion of STD cases diagnosed in the United States are among adolescents and young adults (*1*), STD clinics participating in SSuN have observed an overall decrease in adolescent and young adult patients across jurisdictions, especially among females. Perhaps females, especially adolescent and young adult females, are seeking STD-related health care services elsewhere (e.g., family planning clinics or primary care practices). A study reported that although adolescents and young adults were more likely to have reported receiving an STD test during the previous 12 months, sexually experienced adolescents and young adults more often received their most recent STD test in a private doctor’s office (64.8%) as opposed to an STD clinic (2.2%) (*22*). In addition, the findings presented in this report are consistent with a 2014 study that examined trends in use of STD services among females during 2006–2010 and 2012 and determined that fewer adolescent females aged 15–19 years reported using public clinics for STD-related health services (*23*). Finally, ACA might have eased some cost-related barriers to accessing health care by including a stipulation allowing children of insurance policy holders to remain on their plans until age 26 years (*16*). Thus, adolescents and young adults who once sought care at STD clinics might be obtaining STD-related health services from fee-for-service providers elsewhere, or they might not be getting tested.

### Selected STD-Related Health Services

#### HIV Screening

Both USPSTF and CDC recommend routine screening for HIV infection among persons with diagnosed STDs because timely HIV diagnosis can lead to earlier linkage to care, an earlier start of antiretroviral therapy, and less morbidity and subsequent HIV transmission (*24*,*25*). In this study, the majority of patients who received an acute STD diagnosis who were not known to be living with diagnosed HIV infection were screened for HIV close to the time of their STD diagnosis, with notable increases in HIV screening coverage over the study period. Compared with women and MSW, higher HIV screening coverage was noted among MSM, a population that has been disproportionately affected by both STDs and HIV and that accounts for the majority of HIV transmissions in the United States (*26*). HIV screening coverage among SSuN STD clinic patients with diagnosed primary or secondary syphilis was lower than among patients with gonorrhea or chlamydia, a finding that was not expected considering the relatively high comorbidity between HIV and syphilis reported in the literature (*1*). However, one possible explanation might be that patients who received a diagnosis outside of STD clinics might have been screened for HIV with the diagnosing provider but were referred to STD clinics for syphilis treatment. Consequently, HIV screening might not be repeated in the STD clinics because the patients were referred for treatment only.

Although HIV screening coverage increased over time, understanding why patients with an acute STD might not have had any record of an HIV test is important. Variations in HIV screening prevalence might result from multiple factors, including the degree to which providers perceive that their patients are at risk for HIV infection, patients declining HIV screening because they were recently screened, or differences in clinic protocols or policies that define when and for whom HIV screening will be offered (*27*). Additional research that identifies barriers to HIV screening can help program improvement efforts.

HIV screening in STD clinical settings provides a prime opportunity for identifying persons with undiagnosed HIV infection and offering prevention services for persons with negative test results (e.g., HIV pre-exposure prophylaxis [PrEP]). HIV and other STDs share sexual and other risk factors. Moreover, being infected with STDs such as syphilis and gonorrhea can lead to an increased risk for HIV infection. For these reasons, since 1987, U.S. clinical guidelines have recommended routine HIV screening of persons with STDs in all clinical settings. Screening STD patients for HIV identifies substantial numbers of HIV infections. For example, among all sites, states, and independently funded jurisdictions receiving CDC support for HIV screening in 2011, STD clinics conducted 19% of tests performed in health care settings and identified 26% of all new HIV cases (*28*). Guidelines recommend HIV PrEP for persons with diagnosed STDs (*29*), and STD clinical settings are appropriate settings to screen for, initiate, and monitor PrEP use and support its expansion among populations at high risk for HIV infection (*30*). Low rates of HIV screening indicate missed opportunities for linking HIV-negative STD patients to HIV PrEP and other effective interventions for both HIV prevention and care.

#### Chlamydia Screening Among Females Aged 15–24 Years

If untreated, chlamydial infections can lead to serious reproductive health sequalae among females, including pelvic inflammatory disease (PID), chronic pelvic pain, infertility, and life-threatening ectopic pregnancy. Because chlamydial infections are largely asymptomatic among females, screening is a principal component of comprehensive chlamydia prevention and control activities and can reduce the incidence of PID (*31*). This analysis demonstrates that a substantial percentage of females aged 15–24 years seeking care in STD clinics were screened for chlamydia at least once during a calendar year, indicating that STD clinics are an effective setting for providing chlamydia screening for adolescent and young female patients. The percentage screened was far higher than data collected regarding sexually active females enrolled in commercial and Medicaid health plans, with <60% of sexually active women screened annually (*32*). Screening rates did not appear to vary by race/ethnicity among this study population, although screening rates were higher among females aged 20–24 years than among females aged 15–19 years. Among females screened, chlamydia positivity was higher among those aged 15–19 years than among those aged 20–24 years, which highlights the importance of screening and prevention education among this population. Because of the high risk for reinfection, rescreening for reinfection is recommended 3 months after treatment (*11*). In this analysis, approximately 15% of females were rescreened 8–16 weeks after a positive test result. Rescreening fluctuated year to year but the overall trend was consistently suboptimal. The findings of higher positivity at rescreening compared with initial screening might be due to numerous factors, including high community prevalence, re-exposure to untreated sexual partners, and treatment compliance issues (*33*,*34*). Rescreening also might have been focused on the adolescent and young females who were identified as having a higher risk for reinfection, thus overestimating the prevalence of reinfection. Nonetheless, this study highlights the need to raise awareness of implementing rescreening for chlamydia in these clinical settings to detect reinfection and reduce the sequelae of chlamydia. 

Although overall annual chlamydia screening coverage was high, the decreases in the number of young females screened for chlamydia is notable. First, ACA expanded access to health insurance for certain adolescents and young adults beginning in 2014, offering opportunities for them to access preventive services, including chlamydia screening, from different types of health care providers. However, this cannot entirely explain observed trends, especially because the decreases were observed before ACA was fully implemented. Second, funding for public health has been declining, resulting in budget and staffing reductions and closures of clinics providing STD services (*4*)*.* Third, the decrease in female clinic patients is coupled with an increase in the number of MSM seeking services in these clinics. Thus, a shift in clinical services, forced by limited resources, might have transitioned to providing essential STD clinic services, including HIV preventive services (e.g., PrEP and linkage to care among patients living with HIV). Because of the substantial decrease in females seeking care in STD clinics, further study is warranted to ensure they are still accessing health care and are being screened and rescreened as recommended for chlamydia and other STDs.

#### Extragenital (Rectal and Pharyngeal) Chlamydia and Gonorrhea Screening Among MSM

Extragenital gonococcal and chlamydial infections often are asymptomatic and can be readily passed to an uninfected partner, contributing to an increased risk for HIV acquisition or transmission (*35*)*.* During the study period, the percentage of MSM screened for rectal and pharyngeal gonorrhea and chlamydia increased modestly; however, with the substantial increases in the number of MSM seeking care in STD clinics, the crude number of MSM screened for either pathogen doubled. 

Increases in diagnoses of extragenital chlamydial and gonococcal infections were also observed. Increases in disease incidence might account for this, although other explanations, such as increases in disease detection and increased screening, are possible. First, in 2010, STD treatment guidelines recommended using NAATs to routinely screen for rectal and pharyngeal chlamydial and gonococcal infections among MSM (*36*). NAATs have demonstrated higher sensitivity and specificity, compared with culture, for detecting extragenital infections (*37*). Use of these more sensitive and specific assays has led to an increased yield in terms of case detection (*38*,*39*). Second, since 2010, STD clinics have increased their capacity for performing extragenital NAATs (i.e., shifting from culture-based testing) (*40*,*41*). Third, increases in diagnoses of extragenital STDs might reflect increases in HIV PrEP adoption because PrEP practice guidelines include quarterly STD screening (*42*). The percentage of diagnosed extragenital infections observed in this study underscores the importance of continuing to integrate screening and testing into clinical practice. Because these infections often are asymptomatic, identifying and treating extragenital infections are especially vital in attempting to control chlamydia and gonorrhea among MSM.

## Limitations

The findings in this report are subject to at least seven limitations. First, estimates of unique patients, patient visits, and use of selected STD-related health services in the 14 clinics are not representative of all U.S. STD clinics or of other clinical and health care settings that serve populations at risk. Second, classification of sexual orientation and sex of sex partners was based on patient self-reported sex/gender identity or reporting of sex of sex partners, which might have led to misclassification. Third, although participating jurisdictions followed standardized protocols for abstracting data from electronic medical records and sending data to CDC, the data presented were not validated against clinics’ complete medical records. In addition, medical and laboratory records are inherently complex and potentially susceptible to errors or omissions. Fourth, patients might have had STD or HIV screening at another health care facility or might have been offered screening but refused, indicating that these STD-related health measures are minimum estimates. Fifth, symptom status was not uniformly collected and screening might not be differentiated from diagnostic testing. This might have resulted in an overestimate of screening coverage. Sixth, because treatment data were not available, the analysis of chlamydia rescreening coverage was based on the 3 months after the initial positive chlamydia testing visit. In addition, guidelines also suggest that if rescreening at 3 months is not possible, clinicians should retest in the 12-month period after initial treatment. Hence, the rescreening rates in this analysis are more conservative because it was not possible to look across a 12-month time frame. Finally, regarding extragenital screening, a clinical history of sexual exposure by anatomic site was not collected; therefore, certain MSM included in the denominators of this metric might not have been exposed at a specified anatomic site and might not have been candidates for extragenital screening. Consequently, the percentage screened for extragenital infections is likely an underestimate of screening coverage.

## Conclusion

Ensuring that all persons receive quality HIV and STD prevention and treatment services is a vital part of an effective public health response to increasing STD rates (*1*). Although the majority of STDs are diagnosed outside of public STD clinics, publicly funded clinics remain an important safety-net provider of STD-related health services. These clinics provide important STD-related health services, including timely identification and treatment of curable STDs, to patient populations at risk for the consequences of STD and HIV infections. In addition, a portion of these populations continues to use safety-net services because of a need to keep STD testing private, perception of expertise of STD clinic providers, or perception of inclusivity for sexual minorities (*12*).

STD clinics have a principal part in an effective public health care system by focusing resources on priority problems and populations. An example of this is the implementation of express STD testing models for triaging patients to identify those who need to see a medical provider for a comprehensive examination versus asymptomatic patients who can just have laboratory samples submitted for STD testing (*43*). Ongoing sentinel surveillance across STD clinics can help public health partners better understand the needs of the populations that the clinics serve.

Although STD clinics continue to screen adolescent and young females for chlamydial infection at a high rate, a limited percentage was rescreened after a chlamydia diagnosis, resulting in missed opportunities for enhancing service provision. The asymptomatic nature of extragenital chlamydia and gonorrhea, in conjunction with a high prevalence identified among this clinic population, further supports the need to continue screening all anatomic sites of exposure among MSM. With the increasing incidence of STDs and renewed focus on ending the HIV epidemic at the national level, STD clinics are well positioned to offer vital care and prevention services to populations at risk, including MSM, adolescent and young females, and sexual/gender identity minorities. Moreover, describing the trends in health services provided in STD clinics can help develop a better understanding of the clinics’ role and capacity for providing STD- and HIV-related health services as well as opportunities for program improvement.

A core component of Ending the HIV Epidemic: A Plan for America is to provide HIV diagnoses for all persons with HIV infection as early as possible (*44*). HIV screening is an essential service that STD clinics can provide often and systematically to their patients, particularly those with diagnosed STDs. In addition to early diagnosis, the national plan also aims to improve the quality of life for persons living with HIV infection through effective treatment to achieve and maintain viral suppression. Studies have reported the benefits of rapid initiation of antiretroviral therapy (i.e., same day of diagnosis) as a strategy for improving clinical outcomes and possibly decreasing transmission events (*45*–*47*). Increases in the numbers of MSM seeking care at STD clinics in this sentinel surveillance system indicate that these settings are poised to be important clinical partners in linking patients to high-impact HIV preventive services and to facilitate initiation or resumption of treatment among persons living with diagnosed HIV infection.

## References

[1] CDC. Sexually transmitted disease surveillance 2018. Atlanta, GA: US Department of Health and Human Services, CDC, 2019. https://www.cdc.gov/std/stats18/STDSurveillance2018-full-report.pdf

[2] Chesson HW, Spicknall IH, Bingham A, The estimated direct lifetime medical cost of sexually transmitted infections acquired in the United States in 2018. Sex Transm Dis 2021;48:215–21. 10.1097/OLQ.0b013e318285c6d2 33492093 PMC10684254

[3] Himmelstein DU, Woolhandler S. Public health’s falling share of US health spending. Am J Public Health 2016;106:56–7. 10.2105/AJPH.2015.302908 26562115 PMC4695931

[4] Leichliter JS, Heyer K, Peterman TA, US public sexually transmitted disease clinical services in an era of declining public health funding: 2013–14. Sex Transm Dis 2017;44:505–9. 10.1097/OLQ.0000000000000629 28703733 PMC5642112

[5] National Coalition of STD Directors. Fact sheet: STD program capacity and preparedness in the United States: results of a national survey, 2009. Washington, DC: National Coalition of STD Directors. https://www.ncsddc.org/wp-content/uploads/2019/10/Fact-Sheet-STD-Program-Capacity-and-Preparedness-in-the-United-States-Re....pdf

[6] National Association of County and City Health Officials. Local health department job losses and program cuts: findings from the January 2012 survey. Washington, DC: National Association of County and City Health Officials; 2012. https://foodpoisoningbulletin.com/wp-content/uploads/Research-Brief-Final.pdf

[7] CDC. Data security and confidentiality guidelines for HIV, viral hepatitis, sexually transmitted disease, and tuberculosis programs: standards to facilitate sharing and use of surveillance data for public health action. Atlanta, GA: US Department of Health and Human Services, CDC; 2011. https://www.cdc.gov/nchhstp/programintegration/docs/pcsidatasecurityguidelines.pdf

[8] Owens DK, Davidson KW, Krist AH, ; US Preventive Services Task Force. Screening for HIV infection: US Preventive Services Task Force recommendation statement. JAMA 2019;321:2326–36. 10.1001/jama.2019.6587 31184701

[9] CDC. De-duplication guidance for gonorrhea and chlamydia laboratory reports. Atlanta, GA: US Department of Health and Human Services, CDC; 2016. https://www.cdc.gov/std/laboratory/de-duplication-guidance-june2016.pdf

[10] LeFevre ML; US Preventive Services Task Force. Screening for chlamydia and gonorrhea: U.S. Preventive Services Task Force recommendation statement. Ann Intern Med 2014;161:902–10. 10.7326/M14-1981 25243785

[11] Workowski KA, Bachmann LH, Chan PA, Sexually transmitted infections treatment guidelines, 2021. MMWR Recomm Rep 2021;70(No. RR-4). 10.15585/mmwr.rr7004a1 34292926 PMC8344968

[12] Hoover KW, Parsell BW, Leichliter JS, Continuing need for sexually transmitted disease clinics after the Affordable Care Act. Am J Public Health 2015;105(Suppl 5):S690–5. 10.2105/AJPH.2015.302839 26447908 PMC4627523

[13] Golden MR, Kerndt PR. Improving clinical operations: can we and should we save our STD clinics? Sex Transm Dis 2010;37:264–5. 10.1097/OLQ.0b013e3181d5e01e 20182405

[14] Pang SA. Despite STDs surging to 20-year high, Congress cuts FY ’17 STD funding [Press release]. Washington, DC: National Coalition of STD Directors; 2017. https://www.ncsddc.org/despite-stds-surging-to-20-year-high-congress-cuts-fy-17-std-funding

[15] Lin F, Lasry A, Sansom SL, Wolitski RJ. Estimating the impact of state budget cuts and redirection of prevention resources on the HIV epidemic in 59 California local health departments. PLoS One 2013;8:e55713. 10.1371/journal.pone.0055713 23520447 PMC3592871

[16] Obama B. United States health care reform: progress to date and next steps. JAMA 2016;316:525–32. 10.1001/jama.2016.9797 27400401 PMC5069435

[17] Skopec L, Holahan J, Elmendorf C. Changes in health insurance coverage 2013–2016: Medicaid expansion states lead the way. Princeton, NJ: Robert Wood Johnson Foundation; 2018. https://www.rwjf.org/content/dam/farm/reports/issue_briefs/2018/rwjf448230

[18] Mettenbrink C, Al-Tayyib A, Eggert J, Thrun M. Assessing the changed landscape of sexual health clinic service after the implementation of the Affordable Care Act. Sex Transm Dis 2015;42:725–30. 10.1097/OLQ.0000000000000375 26562704

[19] Stephens SC, Cohen SE, Philip SS, Bernstein KT. Insurance among patients seeking care at a municipal sexually transmitted disease clinic: implications for health care reform in the United States. Sex Transm Dis 2014;41:227–32. 10.1097/OLQ.0000000000000109 24622632

[20] White Hughto JM, Reisner SL, Pachankis JE. Transgender stigma and health: a critical review of stigma determinants, mechanisms, and interventions. Soc Sci Med 2015;147:222–31. 10.1016/j.socscimed.2015.11.010 26599625 PMC4689648

[21] Wansom T, Guadamuz TE, Vasan S. Transgender populations and HIV: unique risks, challenges and opportunities. J Virus Erad 2016;2:87–93. 10.1016/S2055-6640(20)30475-1 27482441 PMC4965251

[22] Cuffe KM, Newton-Levinson A, Gift TL, McFarlane M, Leichliter JS. Sexually transmitted infection testing among adolescents and young adults in the United States. J Adolesc Health 2016;58:512–9. 10.1016/j.jadohealth.2016.01.002 26987687

[23] Haderxhanaj LT, Gift TL, Loosier PS, Cramer RC, Leichliter JS. Trends in receipt of sexually transmitted disease services among women 15 to 44 years old in the United States, 2002 to 2006–2010. Sex Transm Dis 2014;41:67–73. 10.1097/OLQ.0000000000000058 24335746 PMC5795626

[24] Moyer VA; US Preventive Services Task Force. Screening for HIV: U.S. Preventive Services Task Force recommendation statement. Ann Intern Med 2013;159:51–60. 10.7326/0003-4819-159-1-201307020-00645 23698354

[25] Branson BM, Handsfield HH, Lampe MA, Revised recommendations for HIV testing of adults, adolescents, and pregnant women in health-care settings. MMWR Recomm Rep 2006;55(No. RR-14). 16988643

[26] Hall HI, Song R, Tang T, HIV trends in the United States: diagnoses and estimated incidence. JMIR Public Health Surveill 2017;3:e8. 10.2196/publichealth.7051 28159730 PMC5315764

[27] Llata E. HIV testing coverage among STD clinic patients diagnosed with sexually transmitted diseases, STD Surveillance Network, 2017. Presented at the National HIV Prevention Conference, Atlanta, GA; March 18–21, 2019.

[28] Seth P, Wang G, Sizemore E, Hogben M. HIV testing and HIV service delivery to populations at high risk attending sexually transmitted disease clinics in the United States, 2011–2013. Am J Public Health 2015;105:2374–81. 10.2105/AJPH.2015.302778 26378854 PMC4605158

[29] Owens DK, Davidson KW, Krist AH, ; US Preventive Services Task Force. Preexposure prophylaxis for the prevention of HIV infection: US Preventive Services Task Force recommendation statement. JAMA 2019;321:2203–13. 10.1001/jama.2019.6390 31184747

[30] Cohen SE, Vittinghoff E, Bacon O, High interest in preexposure prophylaxis among men who have sex with men at risk for HIV infection: baseline data from the US PrEP demonstration project. J Acquir Immune Defic Syndr 2015;68:439–48. 10.1097/QAI.0000000000000479 25501614 PMC4334721

[31] Oakeshott P, Kerry S, Aghaizu A, Randomised controlled trial of screening for *Chlamydia trachomatis* to prevent pelvic inflammatory disease: the POPI (prevention of pelvic infection) trial. BMJ 2010;340:c1642. 10.1136/bmj.c1642 20378636 PMC2851939

[32] National Committee for Quality Assurance. The state of healthcare quality 2018. Washington, DC: National Committee for Quality Assurance, 2018. https://www.ncqa.org/hedis/measures/chlamydia-screening-in-women/

[33] Gaydos CA, Wright C, Wood BJ, Waterfield G, Hobson S, Quinn TC. *Chlamydia trachomatis* reinfection rates among female adolescents seeking rescreening in school-based health centers. Sex Transm Dis 2008;35:233–7. 10.1097/OLQ.0b013e31815c11fe 18490866 PMC2664683

[34] Cha S, Newman DR, Rahman M, Peterman TA. High rates of repeat chlamydial infections among young women—Louisiana, 2000–2015. Sex Transm Dis 2019;46:52–7. 10.1097/OLQ.0000000000000906 30148756 PMC6291349

[35] Fleming DT, Wasserheit JN. From epidemiological synergy to public health policy and practice: the contribution of other sexually transmitted diseases to sexual transmission of HIV infection. Sex Transm Infect 1999;75:3–17. 10.1136/sti.75.1.3 10448335 PMC1758168

[36] Workowski KA, Berman S; CDC. Sexually transmitted diseases treatment guidelines, 2010. MMWR Recomm Rep 2010;59(No. RR-12). Erratum in: MMWR Morb Mortal Wkly Rep 2011;60:18. 21160459

[37] . Chan PA, Robinette A, Montgomery M, Extragenital infections caused by *Chlamydia trachomatis* and *Neisseria gonorrhoeae*: a review of the literature. Infect Dis Obstet Gynecol 2016;2016:5758387. 10.1155/2016/5758387 27366021 PMC4913006

[38] Barbee LA, Dombrowski JC, Kerani R, Golden MR. Effect of nucleic acid amplification testing on detection of extragenital gonorrhea and chlamydial infections in men who have sex with men sexually transmitted disease clinic patients. Sex Transm Dis 2014;41:168–72. 10.1097/OLQ.0000000000000093 24521722

[39] Cornelisse VJ, Chow EP, Huffam S, Increased detection of pharyngeal and rectal gonorrhea in men who have sex with men after transition from culture to nucleic acid amplification testing. Sex Transm Dis 2017;44:114–7. 10.1097/OLQ.0000000000000553 27984552

[40] Anschuetz GL, Paulukonis E, Powers R, Asbel LE. Extragenital screening in men who have sex with men diagnoses more chlamydia and gonorrhea cases than urine testing alone. Sex Transm Dis 2016;43:299–301. 10.1097/OLQ.0000000000000435 27100766

[41] Patton ME, Kidd S, Llata E, Extragenital gonorrhea and chlamydia testing and infection among men who have sex with men—STD Surveillance Network, United States, 2010–2012. Clin Infect Dis 2014;58:1564–70. 10.1093/cid/ciu184 24647015 PMC4666527

[42] Grant RM, Anderson PL, McMahan V, ; iPrEx study team. Uptake of pre-exposure prophylaxis, sexual practices, and HIV incidence in men and transgender women who have sex with men: a cohort study. Lancet Infect Dis 2014;14:820–9. 10.1016/S1473-3099(14)70847-3 25065857 PMC6107918

[43] Whitlock GG, Gibbons DC, Longford N, Harvey MJ, McOwan A, Adams EJ. Rapid testing and treatment for sexually transmitted infections improve patient care and yield public health benefits. Int J STD AIDS 2018;29:474–82. 10.1177/0956462417736431 29059032 PMC5844454

[44] US Department of Health and Human Services. What is ending the HIV epidemic in the U.S.? Rockville, MD: US Department of Health and Human Services; 2021. https://www.hiv.gov/federal-response/ending-the-hiv-epidemic/overview

[45] Coffey S, Bacchetti P, Sachdev D, RAPID antiretroviral therapy: high virologic suppression rates with immediate antiretroviral therapy initiation in a vulnerable urban clinic population. AIDS 2019;33:825–32. 10.1097/QAD.0000000000002124 30882490 PMC7029629

[46] Koenig SP, Dorvil N, Dévieux JG, Same-day HIV testing with initiation of antiretroviral therapy versus standard care for persons living with HIV: a randomized unblinded trial. PLoS Med 2017;14:e1002357. 10.1371/journal.pmed.1002357 28742880 PMC5526526

[47] Ford N, Migone C, Calmy A, Benefits and risks of rapid initiation of antiretroviral therapy. AIDS 2018;32:17–23. 10.1097/QAD.0000000000001671 29112073 PMC5732637

